# Crystal structure and Hirshfeld surface analysis of poly[[tetra­aqua­(μ-1,3,4,7,8,10,12,13,16,17,19,22-dodeca­aza­tetra­cyclo­[8.8.4.1^3,17^.1^8,12^]tetra­cosane-5,6,14,15,20,21-hexaonato)iron(IV)dilithium] tetra­hydrate]

**DOI:** 10.1107/S2056989023008587

**Published:** 2023-10-19

**Authors:** Maksym O. Plutenko, Sergiu Shova, Vadim A. Pavlenko, Irina A. Golenya, Igor O. Fritsky

**Affiliations:** a Department of Chemistry, National Taras Shevchenko University, Volodymyrska Street 64, 01601 Kyiv, Ukraine; b PetruPoni Institute of Macromolecular Chemistry, Aleea Gr. Ghica Voda, 41 A, Iasi 700487, Romania; c Innovation development center ABN, Pirogov str.2/37, 01030 Kiev, Ukraine; University of Neuchâtel, Switzerland

**Keywords:** crystal structure, iron(IV) complex, clathrochelate, template reaction, macrocyclic ligand, hydrazide-based ligand, Hirshfeld surface analysis

## Abstract

The title compound, [FeLi_2_(C_12_H_12_N_12_O_6_)(H_2_O)_4_]·4H_2_O, consists of iron complex anions, lithium cations and water mol­ecules. The complex anion shows a clathrochelate topology. The coordination geometry of the Fe^IV^ centre is inter­mediate between a trigonal prism and a trigonal anti­prism. In the crystal, the complex anions are connected through two Li cations into dimers, which are connected by Li—O bonds, forming infinite chains along the *b-*axis direction.

## Abstract 2

The title compound was obtained as a result of a template reaction between oxalohydrazide, formaldehyde and iron(III) chloride in the presence of atmospheric O_2_. The complex anion of the title compound reveals clathrochelate topology and includes an Fe^IV^ metal centre. In the crystal, the complex anions are connected through two Li cations into dimers, which are connected by Li—O bonds, forming infinite chains along the *b-*axis direction.

## 1. Chemical context

In 2017, a series of unprecedentedly stable iron(IV) complexes was described (Tomyn *et al.*, 2017 ▸). The substances can be obtained by a one-pot template reaction between iron(III) salts, oxalodihydrazide and formaldehyde in the presence of atmospheric oxygen in alkaline aqueous media. All complexes possess the clathrochelate topology with very similar geometric parameters for the Fe^IV^ atom but different crystal packings. Further studies showed that these compounds are promising redox catalysts for photochemical water splitting (Shylin *et al.*, 2019 ▸) and can be used as building blocks for obtaining new metal–organic frameworks (Xu *et al.*, 2020*a* ▸,*b* ▸, 2020 ▸). 
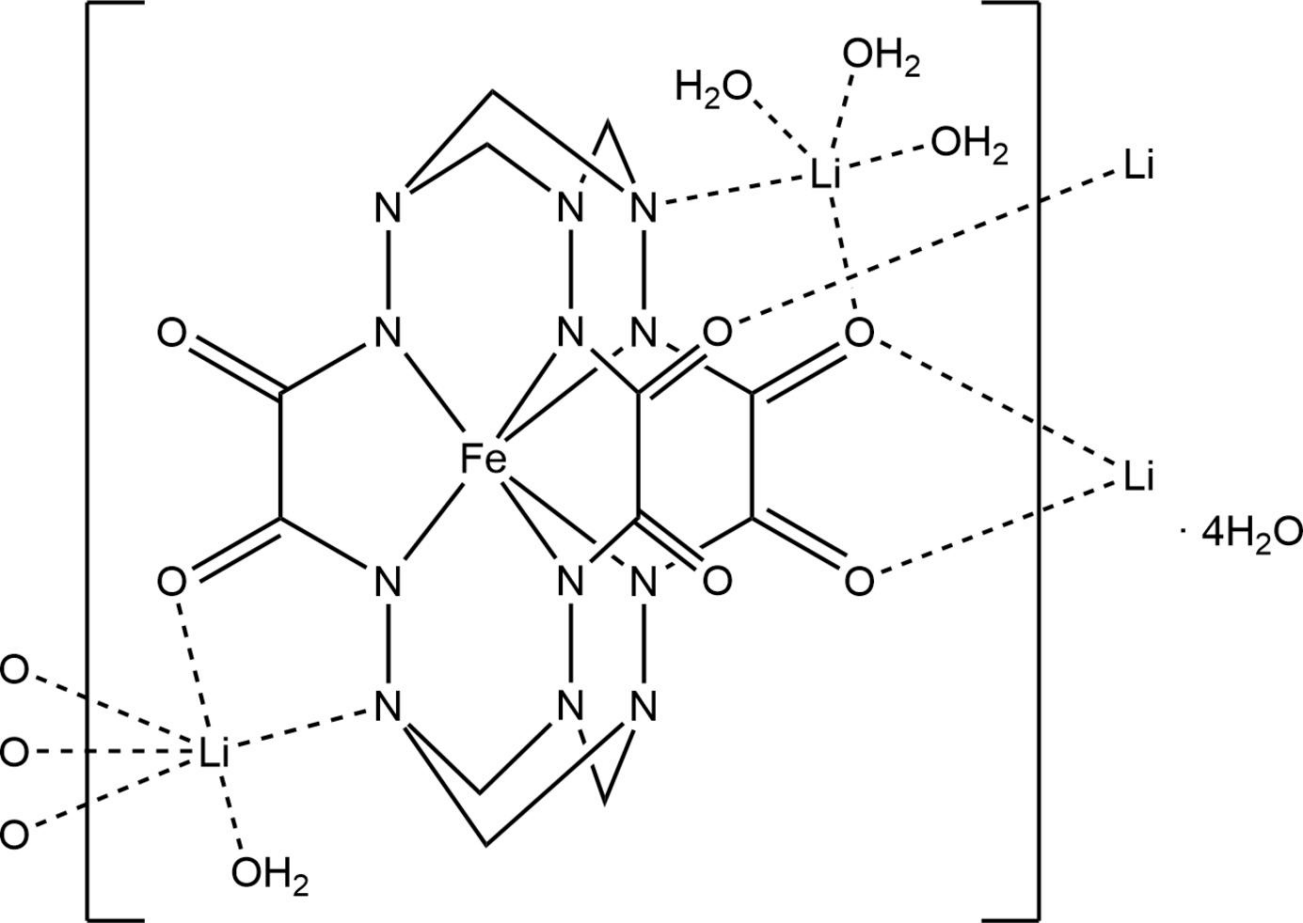


**Figure 1:**
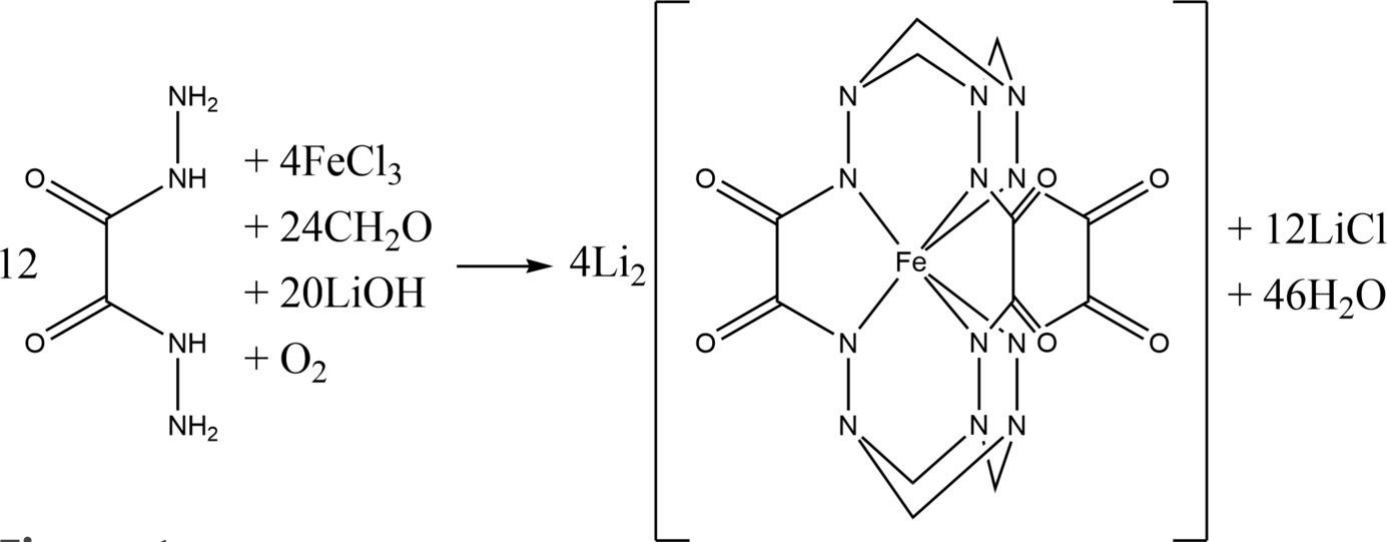
The synthesis of the title compound.

Here, we report the synthesis, crystal structure and Hirshfeld surface analysis of the title compound Li_2_[Fe*L*]·8H_2_O (H_6_ *L* = (1s,3s,8s,10s,12s,17s)-1,3,4,7,8,10,12,13,16,17,19,22- dodeca­aza­tetra­cyclo­[8.8.4.1^3,17^.1^8,12^]tetra­cosane-5,6,14,15,20,21-hexa­one) (**1**) obtained as a result of a template reaction between oxalohydrazide, formaldehyde and iron(III) chloride in the presence of atmospheric oxygen (Fig. 1 ▸). Thus, the present work is devoted to the further study of the synthetic approach proposed by Tomyn and co-workers (Tomyn *et al.*, 2017 ▸). This work is also a continuation of our research into template aldehyde–hydrazide inter­actions in the presence of 3*d* metal ions (Plutenko *et al.*, 2021*a* ▸,*b* ▸).

## 2. Structural commentary

**Figure 2:**
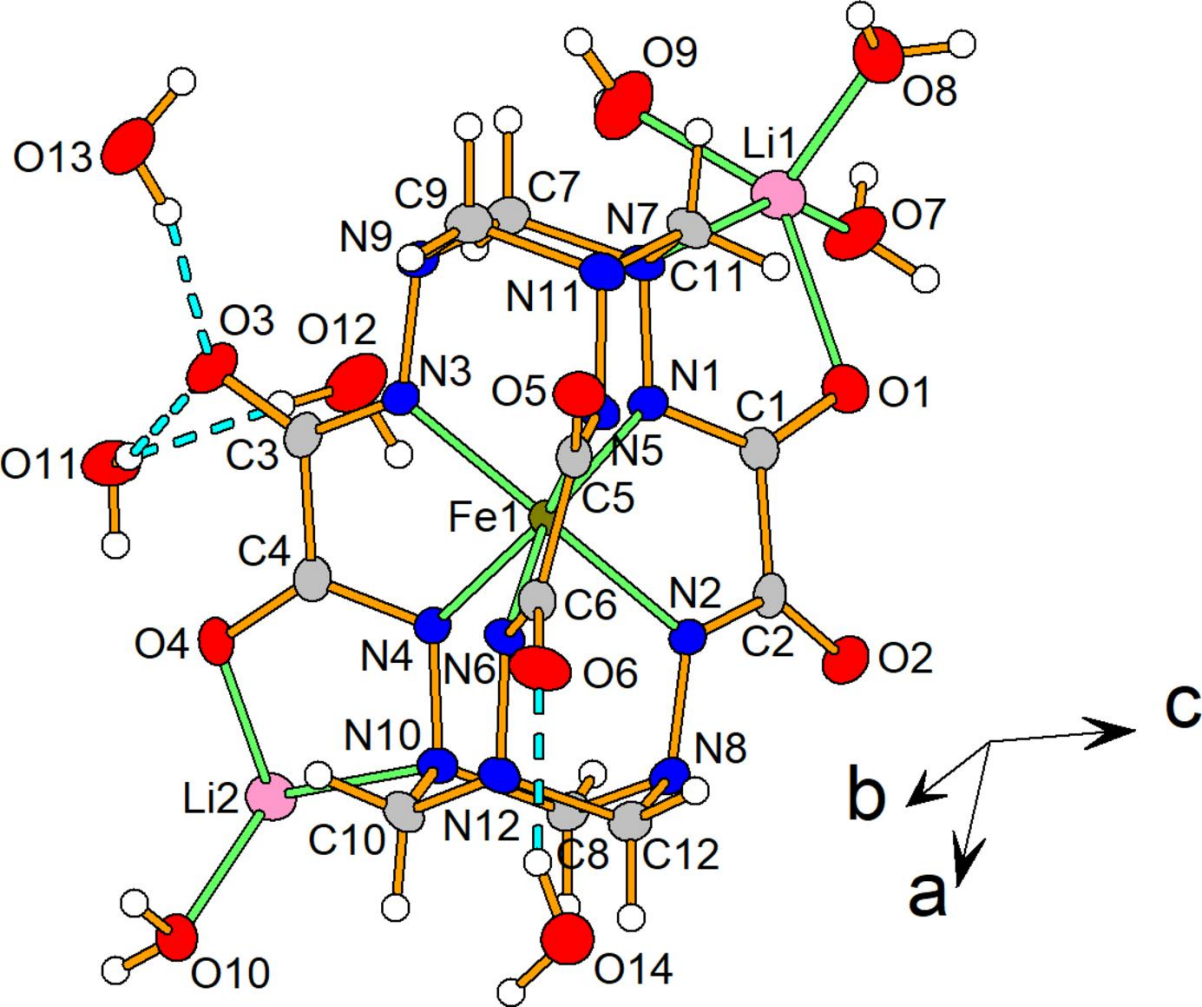
The asymmetric unit of the title compound with displacement ellipsoids shown at the 50% probability level.

**Figure 3:**
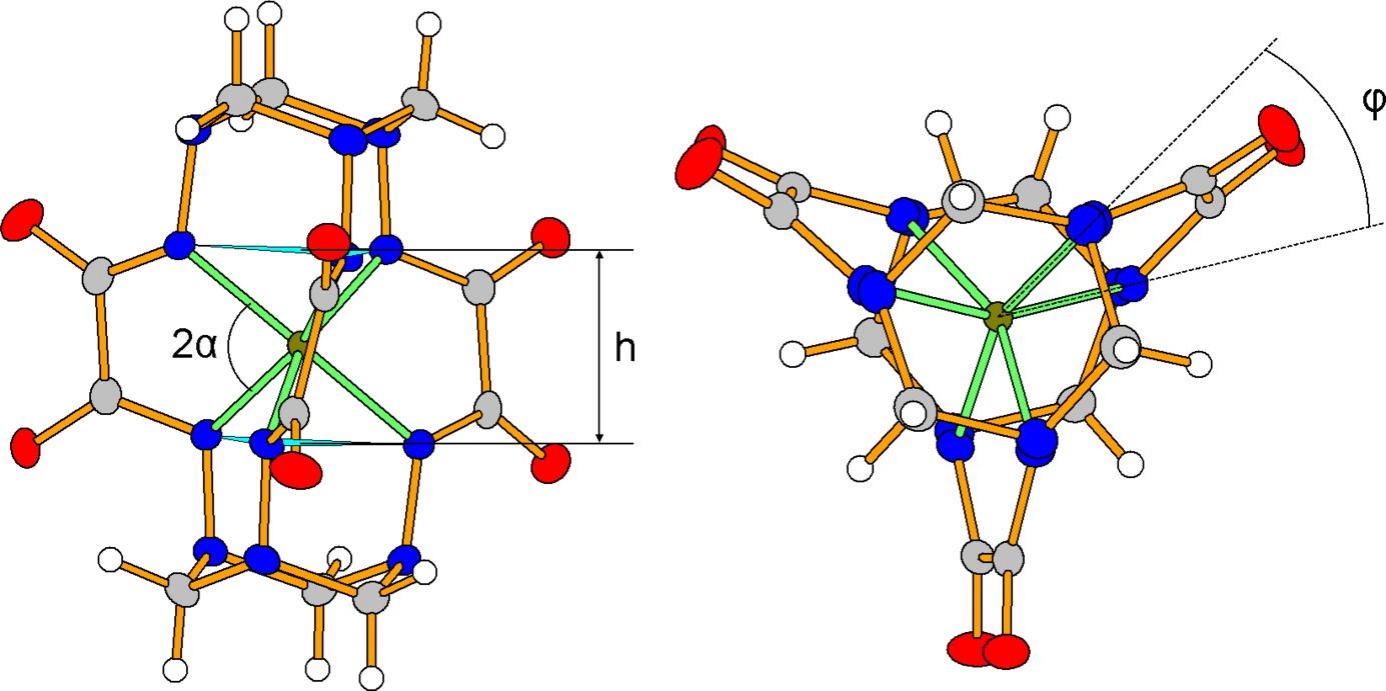
TP–TAP distortion of the FeN_6_ polyhedron in the complex anion.

The title compound crystallizes in the *C*2/*c* space group. The unit cell contains eight complex anions [Fe*L*]^2−^, 16 lithium cations and 64 water mol­ecules (Fig. 2 ▸). The coord­ination geometry of the Fe^IV^ centre (Fig. 3 ▸) is inter­mediate between a trigonal prism (TP, distortion angle φ = 0°) and a trigonal anti­prism (TAP, distortion angle φ = 60°); the distortion angle φ average value being 33.04 (5)°, which is quite close to those of the earlier published Fe^IV^ clathrochelates (28.0–31.9°) (Tomyn *et al.*, 2017 ▸).

The Fe1—N bond distances are in the range 1.9340 (17)–1.9572 (15) Å (Table 1 ▸). The N⋯N separations in the hydrazide apical groups vary from 2.670 (2) to 2.701 (3) Å. The height of the coordination polyhedron *h* is equal to 2.3557 (13) Å. The bite angle α (half of the chelate N—Fe—N′ angle) is equal to 40.53 (4)°, the chelate N—Fe—N′ angles being in the range 80.29 (6)–80.87 (6)°. Thus, all geometric parameters of the Fe^IV^ coordination polyhedron are close to those of the earlier published Fe^IV^ clathrochelates (Tomyn *et al.*, 2017 ▸).

## 3. Supra­molecular features

**Figure 4:**
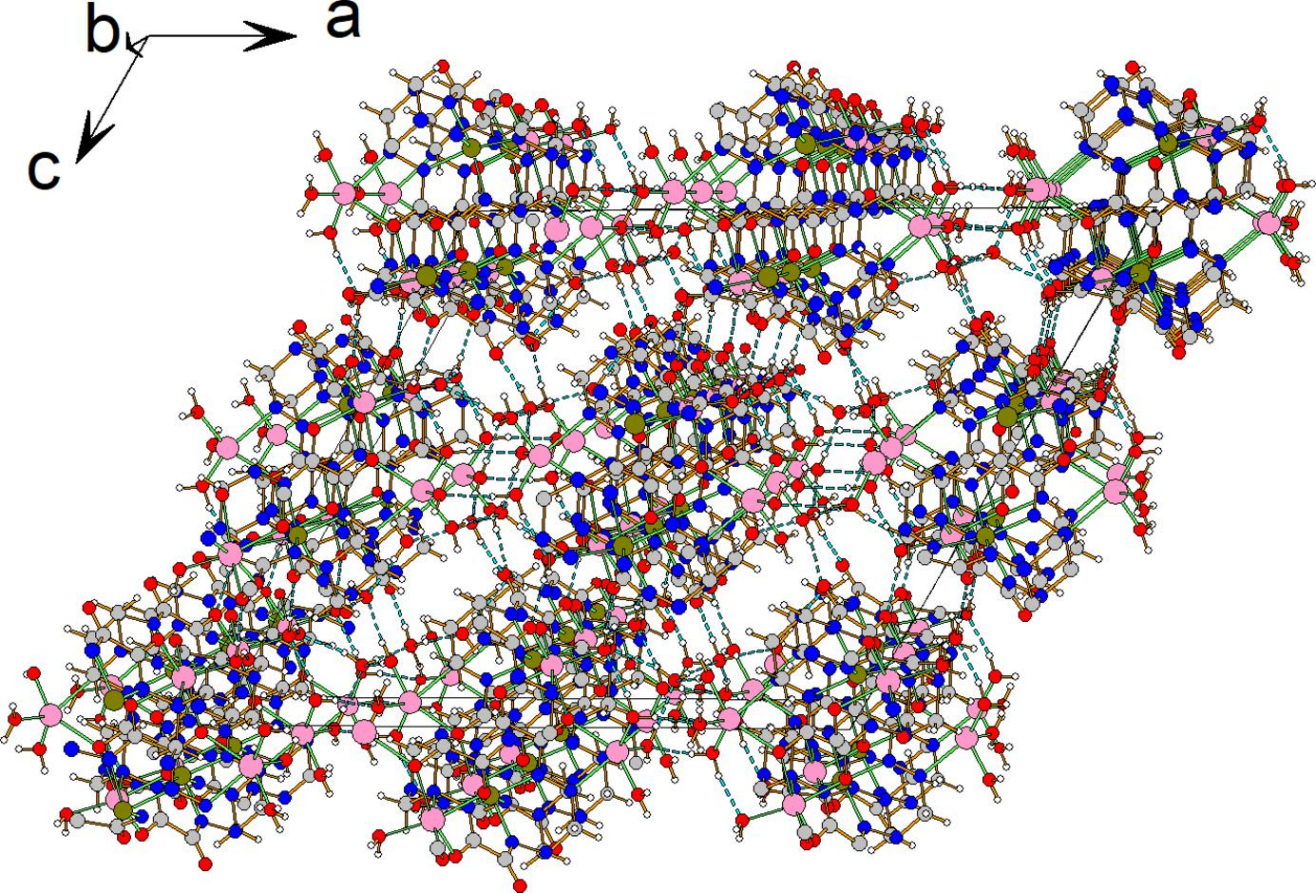
Crystal packing of the title compound. Hydrogen bonds are indicated by dashed lines.

It is important to note that the [Fe*L*]^2−^ complex anion is chiral. Both stereoisomers of the complex cation are included in the crystal packing, thus, **1** is a racemate. In the crystal, both chiral isomers are connected through two Li cations (by O4⋯Li2, N10⋯Li2, O1⋯Li2 and O2⋯Li2 inter­actions), forming a racemic dimer {Li_2_[Fe*L*]_2_}^2−^. Such dimers are connected by Li2⋯O5 inter­actions, forming continuous chains along the *b-*axis direction (Fig. 4 ▸).

In addition, the crystal structure is consolidated by an extensive system of hydrogen bonds (Table 2 ▸). Based on the results of recent studies (Lobato *et al.*, 2021 ▸), the distance of 2.14 Å was used as a criterion for the demarcation of O—H⋯O hydrogen bonds and O⋯H van der Waals inter­actions. According to this criterion, 14 O⋯H contacts were identified as hydrogen bonds.

## 4. Hirshfeld analysis

**Figure 5:**
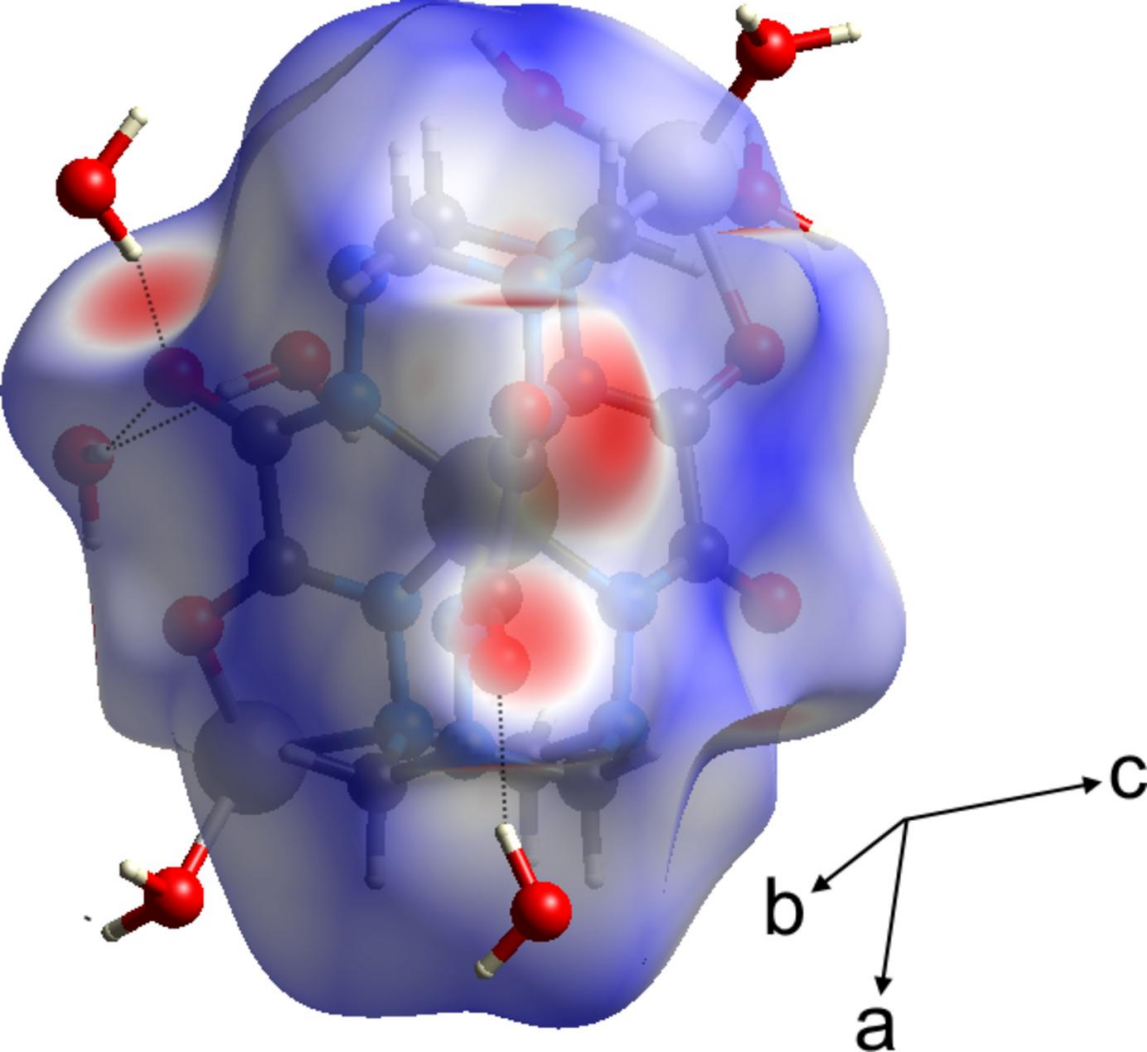
The Hirshfeld surfaces of the complex anion mapped over *d*_norm_.

The Hirshfeld surface analysis (Spackman & Jayatilaka, 2009 ▸) and the associated two-dimensional fingerprint plots (McKinnon *et al.*, 2007 ▸) were performed with *CrystalExplorer17* (Turner *et al.*, 2017 ▸). The Hirshfeld surfaces of the [Fe*L*]^2−^ complex anion are colour-mapped with the normalized contact distance (*d*_norm_) from red (distances shorter than the sum of the van der Waals radii) through white to blue (distances longer than the sum of the van der Waals radii). The Hirshfeld surface of the title compound mapped over *d*_norm_ is shown in Fig. 5 ▸. According to the Hirshfeld surface, the most noticeable inter­molecular inter­action are Li⋯O contacts (O1⋯Li1, N7⋯Li1, O1⋯Li2, O2⋯Li2, O4⋯Li2, O5⋯Li2, N10⋯Li2) and O–H⋯O hydrogen bonds (O10—H00*B*⋯O4, O13—H00*R*⋯O3, O11—H00*O*⋯O3, O14—H00*M*⋯O6, O10—H00*A*⋯O6).

**Figure 6:**
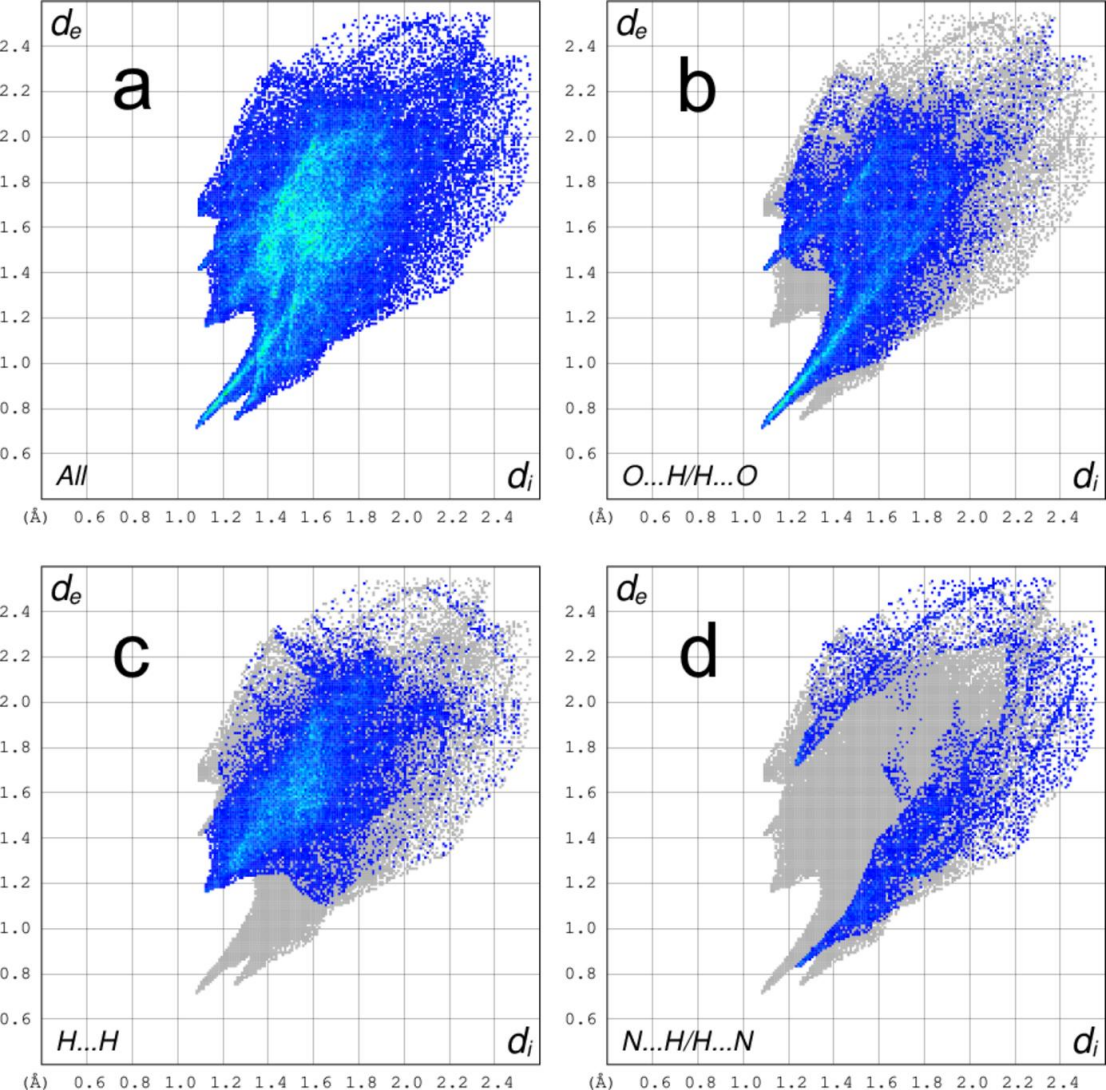
(*a*) Full two-dimensional fingerprint plot of the complex anion and delineated into (*b*) O⋯H/H⋯O (33.3%) (*c*) H⋯H (32.9%) and (*d*) N⋯H/H⋯N (8.9%) contacts.

A fingerprint plot delineated into specific inter­atomic contacts contains information related to specific inter­molecular inter­actions. The blue colour refers to the frequency of occurrence of the (*d*_i_, *d*_e_) pair with the full fingerprint plot outlined in grey. Fig. 6 ▸ shows the two-dimensional fingerprint plot of the sum of the contacts contributing to the Hirshfeld surface. The most significant contributions to the Hirshfeld surface are from O⋯H/H⋯O (33.3%) and H⋯H (32.9%) contacts. In addition, N⋯H/H⋯N (8.9%) is also a highly significant contribution to the total Hirshfeld surface.

## 5. Database survey

A search in the Cambridge Structural Database (CSD version 5.43, update of November 2022; Groom *et al.*, 2016 ▸) resulted in nine hits dealing with hydrazide-based clathrochelates of 3d-metals. There are three structures of Mn^IV^ clathrochelates (Shylin *et al.*, 2021 ▸; Xu *et al.*, 2022 ▸), three structures of Fe^IV^ clathrochelates (Tomyn *et al.*, 2017 ▸) and three hits dealing with Fe^IV^ clathrochelate-based metal-organic frameworks (MOFs). The MOFs reveal a 1D coordination polymer topology: the Fe^IV^ clathrochelate complex anions being connected by Mn^2+^ (Xu *et al.*, 2020*b* ▸) or Cu^2+^ (Xu *et al.*, 2020*a* ▸, 2022 ▸) cations, forming zigzag hetero-bimetallic chains, and being bimetallic helps to understand the link with Mn^2+^ and Cu^2+^.

## 6. Synthesis and crystallization

To a mixture of 0.354 g oxalodihydrazide (3 mmol) and 0.144 g LiOH (6 mmol), 10 ml of FeCl_3_ aqueous solution (1 mmol) were added dropwise. Then an aqueous formaldehyde solution (37% in water, 0.73 ml, 9 mmol) was added. The reaction mixture was stirred for 2 h under slight warming (∼313 K), filtered off, and the solvent removed on a rotary evaporator. The crude product was dissolved in 5 ml of water and left for crystallization by slow diffusion of tetra­hydro­furan vapour. Single crystals suitable for X-ray analysis were obtained after one month. Yield 0.124g (22%). IR (KBr, cm^−1^): 3409 (O—H), 2942 (C—H), 1648 (C=O amide I). Analysis calculated for C_12_H_28_FeLi_2_N_12_O_14_: C 22.73, H 4.45, N 26.51. Found: C 22.79, H 4.36, N 26.73.

## 7. Refinement

Crystal data, data collection and structure refinement details are summarized in Table 3 ▸. The water hydrogen atoms were located in a difference-Fourier map and refined isotropically. Other hydrogen atoms were positioned geometrically and constrained to ride on their parent atoms, with C—H = 0.99 Å, and *U*_iso_(H)= 1.2*U*_eq_(parent atom).

## Supplementary Material

Crystal structure: contains datablock(s) I. DOI: 10.1107/S2056989023008587/tx2074sup1.cif

Structure factors: contains datablock(s) I. DOI: 10.1107/S2056989023008587/tx2074Isup2.hkl

Supporting information file. DOI: 10.1107/S2056989023008587/tx2074Isup3.cdx

CCDC reference: 2298136

Additional supporting information:  crystallographic information; 3D view; checkCIF report

## Figures and Tables

**Table 1:** Selected geometric parameters (Å, °)

Fe1—N1	1.9405 (15)	N5—Fe1—N6	80.87 (8)
Fe1—N2	1.9572 (15)	N1⋯N3	2.688 (3)
Fe1—N3	1.9516 (16)	N1⋯N3	2.672 (3)
Fe1—N4	1.9504 (16)	N3⋯N5	2.673 (2)
Fe1—N5	1.9340 (16)	N2⋯N4	2.689 (2)
Fe1—N6	1.9398 (15)	N2⋯N6	2.701 (3)
N1—Fe1—N2	80.43 (6)	N4⋯N6	2.670 (2)
N3—Fe1—N4	80.29 (6)		

**Table 2:** Hydrogen-bond geometry (Å, °)

*D*—H⋯*A*	*D*—H	H⋯*A*	*D*⋯*A*	*D*—H⋯*A*
O7—H00*E*⋯O10^i^	0.86	1.87	2.715 (2)	167
O7—H00*F*⋯O8^ii^	0.86	2.08	2.921 (2)	164
O8—H00*C*⋯O14^iii^	0.86	1.90	2.759 (2)	175
O8—H00*D*⋯O13^iv^	0.86	1.97	2.812 (2)	167
O9—H00*G*⋯O11^iv^	0.86	1.97	2.827 (2)	176
O9—H00*H*⋯O14^v^	0.86	2.02	2.881 (2)	177
O10—H00*A*⋯O6^vi^	0.87	2.00	2.768 (2)	147
O10—H00*B*⋯O4^vii^	0.87	1.97	2.8301 (18)	178
O11—H00*O*⋯O3	0.86	2.05	2.844 (2)	154
O12—H00*S*⋯O11	0.86	1.97	2.828 (2)	177
O13—H00*Q*⋯O12^iv^	0.86	1.88	2.736 (3)	177
O13—H00*R*⋯O3	0.86	1.93	2.767 (2)	165
O14—H00*M*⋯O6	0.86	1.98	2.784 (2)	155
O14—H00*N*⋯O13^vii^	0.86	2.01	2.867 (2)	176

Symmetry codes: (i) 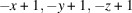 ; (ii) 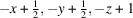 ; (iii) 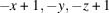 ; (iv) 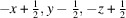 ; (v) 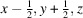 ; (vi) 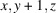 ; (vii) 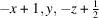 .

**Table 3:** Experimental details

Crystal data	
Chemical formula	[FeLi_2_(C_12_H_12_N_12_O_6_)(H_2_O)_4_]·4H_2_O
*M*_r_	634.19
Crystal system, space group	Monoclinic, *C*2/*c*
Temperature (K)	240
*a*, *b*, *c* (Å)	25.4076 (8), 9.9854 (2), 22.3570 (8)
β (°)	120.265 (5)
*V* (Å^3^)	4899.0 (3)
*Z*	8
Radiation type	Mo *K*α
μ (mm^−1^)	0.71
Crystal size (mm)	0.35 × 0.25 × 0.15

Data collection	
Diffractometer	Xcalibur, Eos
Absorption correction	Multi-scan (*CrysAlis PRO*; Rigaku OD, 2021 ▸)
*T*_min_, *T*_max_	0.856, 1.000
No. of measured, independent and observed [*I* > 2σ(*I*)] reflections	15658, 5611, 4704
*R*_int_	0.028
(sin θ/λ)_max_ (Å^−1^)	0.688

Refinement	
*R*[*F* ^2^ > 2σ(*F* ^2^)], *wR*(*F* ^2^), *S*	0.035, 0.086, 1.05
No. of reflections	5611
No. of parameters	370
No. of restraints	3
H-atom treatment	H-atom parameters constrained
Δρ_max_, Δρ_min_ (e Å^−3^)	0.36, −0.50

Computer programs: *CrysAlis PRO* (Rigaku OD, 2021 ▸), *SHELXT* (Sheldrick, 2015*a* ▸), *SHELXL2018/3* (Sheldrick, 2015*b* ▸) and *OLEX2* (Dolomanov *et al.*, 2009 ▸).

## supplementary crystallographic information

### Crystal data

**Table:**

[FeLi_2_(C_12_H_12_N_12_O_6_)(H_2_O)_4_]·4H_2_O	*F*(000) = 2624
*M**_r_* = 634.19	*D*_x_ = 1.720 Mg m^−^^3^
Monoclinic, *C*2/*c*	Mo *K*α radiation, λ = 0.71073 Å
*a* = 25.4076 (8) Å	Cell parameters from 6976 reflections
*b* = 9.9854 (2) Å	θ = 2.3–29.0°
*c* = 22.3570 (8) Å	µ = 0.71 mm^−^^1^
β = 120.265 (5)°	*T* = 240 K
*V* = 4899.0 (3) Å^3^	Plate, clear dark brown
*Z* = 8	0.35 × 0.25 × 0.15 mm

### Data collection

**Table:**

Xcalibur, Eos diffractometer	4704 reflections with *I* > 2σ(*I*)
Radiation source: fine-focus sealed X-ray tube	*R*_int_ = 0.028
ω scans	θ_max_ = 29.3°, θ_min_ = 1.9°
Absorption correction: multi-scan (CrysAlisPro; Rigaku OD, 2021)	*h* = −31→34
*T*_min_ = 0.856, *T*_max_ = 1.000	*k* = −12→12
15658 measured reflections	*l* = −30→28
5611 independent reflections	3 standard reflections every 100 reflections

### Refinement

**Table:**

Refinement on *F*^2^	3 restraints
Least-squares matrix: full	Hydrogen site location: mixed
*R*[*F*^2^ > 2σ(*F*^2^)] = 0.035	H-atom parameters constrained
*wR*(*F*^2^) = 0.086	*w* = 1/[σ^2^(*F*_o_^2^) + (0.0346*P*)^2^ + 4.922*P*] where *P* = (*F*_o_^2^ + 2*F*_c_^2^)/3
*S* = 1.05	(Δ/σ)_max_ < 0.001
5611 reflections	Δρ_max_ = 0.36 e Å^−^^3^
370 parameters	Δρ_min_ = −0.50 e Å^−^^3^

### Special details

**Table:**

Geometry. All esds (except the esd in the dihedral angle between two l.s. planes) are estimated using the full covariance matrix. The cell esds are taken into account individually in the estimation of esds in distances, angles and torsion angles; correlations between esds in cell parameters are only used when they are defined by crystal symmetry. An approximate (isotropic) treatment of cell esds is used for estimating esds involving l.s. planes.

### Fractional atomic coordinates and isotropic or equivalent isotropic displacement parameters (Å^2^)

**Table:**

	*x*	*y*	*z*	*U*_iso_*/*U*_eq_	
Fe1	0.46624 (2)	0.22908 (3)	0.38076 (2)	0.01262 (8)	
Li1	0.31903 (16)	0.2166 (4)	0.4662 (2)	0.0285 (8)	
Li2	0.51611 (16)	0.6998 (4)	0.36559 (18)	0.0247 (8)	
O1	0.41814 (6)	0.20924 (15)	0.52715 (7)	0.0253 (3)	
O2	0.53645 (6)	0.30502 (15)	0.58348 (7)	0.0235 (3)	
O3	0.34175 (7)	0.45564 (14)	0.22349 (8)	0.0304 (4)	
O4	0.45683 (6)	0.57917 (13)	0.28837 (7)	0.0210 (3)	
O5	0.46493 (6)	−0.13454 (14)	0.30406 (7)	0.0224 (3)	
O6	0.57720 (6)	−0.00286 (15)	0.34614 (8)	0.0271 (3)	
O7	0.32263 (7)	0.33148 (17)	0.53909 (8)	0.0347 (4)	
H00E	0.350462	0.317568	0.581392	0.052*	
H00F	0.291147	0.354733	0.541452	0.052*	
O8	0.28280 (7)	0.04068 (15)	0.46559 (8)	0.0301 (4)	
H00C	0.286765	0.003240	0.502261	0.045*	
H00D	0.272742	−0.023573	0.436200	0.045*	
O9	0.25088 (7)	0.32555 (17)	0.39188 (8)	0.0368 (4)	
H00G	0.222223	0.298288	0.352207	0.055*	
H00H	0.237199	0.398879	0.399040	0.055*	
O10	0.57787 (6)	0.72100 (14)	0.33567 (7)	0.0204 (3)	
H00A	0.578172	0.803427	0.323940	0.031*	
H00B	0.566542	0.676297	0.297930	0.031*	
O11	0.33933 (7)	0.73725 (15)	0.24099 (8)	0.0291 (3)	
H00O	0.349228	0.659621	0.233194	0.044*	
H00P	0.373327	0.779521	0.263440	0.044*	
O12	0.32125 (8)	0.6666 (2)	0.35183 (9)	0.0512 (5)	
H00S	0.326163	0.685011	0.317356	0.077*	
H00T	0.356921	0.676047	0.387626	0.077*	
O13	0.25329 (7)	0.30629 (16)	0.11515 (8)	0.0351 (4)	
H00Q	0.231203	0.261120	0.127006	0.053*	
H00R	0.281584	0.340185	0.153255	0.053*	
O14	0.69993 (7)	0.06720 (16)	0.41220 (8)	0.0307 (4)	
H00M	0.660923	0.069494	0.386320	0.046*	
H00N	0.712163	0.140947	0.403385	0.046*	
N1	0.41463 (7)	0.22279 (16)	0.42162 (8)	0.0162 (3)	
N2	0.52862 (7)	0.24332 (15)	0.47873 (8)	0.0150 (3)	
N3	0.39332 (7)	0.27406 (16)	0.29324 (8)	0.0163 (3)	
N4	0.48454 (7)	0.41507 (15)	0.37156 (8)	0.0148 (3)	
N5	0.44518 (7)	0.04326 (16)	0.35575 (8)	0.0170 (3)	
N6	0.53075 (7)	0.18096 (16)	0.36242 (8)	0.0154 (3)	
N7	0.35514 (7)	0.16379 (17)	0.38745 (8)	0.0190 (3)	
N8	0.58616 (7)	0.30474 (16)	0.50012 (8)	0.0165 (3)	
N9	0.33731 (7)	0.20469 (16)	0.26972 (8)	0.0186 (3)	
N10	0.54501 (7)	0.46721 (15)	0.40384 (8)	0.0162 (3)	
N11	0.38492 (7)	−0.00511 (17)	0.32933 (8)	0.0197 (3)	
N12	0.58700 (7)	0.25184 (16)	0.39290 (8)	0.0171 (3)	
C1	0.44139 (8)	0.22632 (19)	0.49052 (10)	0.0161 (4)	
C2	0.50845 (8)	0.26239 (19)	0.52339 (9)	0.0160 (4)	
C3	0.38748 (9)	0.39944 (19)	0.27093 (9)	0.0184 (4)	
C4	0.44690 (8)	0.47478 (19)	0.31130 (9)	0.0164 (4)	
C5	0.47741 (8)	−0.02467 (19)	0.33310 (9)	0.0165 (4)	
C6	0.53471 (8)	0.05244 (19)	0.34806 (9)	0.0165 (4)	
C7	0.31630 (9)	0.2270 (2)	0.31928 (10)	0.0212 (4)	
H01A	0.274787	0.191914	0.299432	0.025*	
H01B	0.314716	0.323597	0.325917	0.025*	
C8	0.57650 (8)	0.44681 (19)	0.47912 (9)	0.0176 (4)	
H00I	0.552640	0.489220	0.497301	0.021*	
H00J	0.616152	0.491800	0.500196	0.021*	
C9	0.34453 (9)	0.0618 (2)	0.26241 (10)	0.0222 (4)	
H01C	0.361326	0.049488	0.231784	0.027*	
H01D	0.304373	0.018984	0.240460	0.027*	
C10	0.57816 (9)	0.39403 (19)	0.37480 (10)	0.0189 (4)	
H00K	0.555332	0.402670	0.324251	0.023*	
H00L	0.618074	0.436027	0.391778	0.023*	
C11	0.36310 (9)	0.0196 (2)	0.37765 (10)	0.0212 (4)	
H01G	0.392110	−0.019496	0.422652	0.025*	
H01H	0.323982	−0.026181	0.360628	0.025*	
C12	0.61777 (8)	0.2364 (2)	0.46903 (10)	0.0186 (4)	
H01E	0.620862	0.140837	0.480263	0.022*	
H01F	0.659266	0.272030	0.489694	0.022*	

### Atomic displacement parameters (Å^2^)

**Table:**

	*U*^11^	*U*^22^	*U*^33^	*U*^12^	*U*^13^	*U*^23^
Fe1	0.01409 (13)	0.01225 (15)	0.01214 (13)	−0.00028 (10)	0.00707 (11)	−0.00015 (10)
Li1	0.0260 (18)	0.031 (2)	0.032 (2)	−0.0012 (16)	0.0170 (17)	−0.0024 (16)
Li2	0.0267 (18)	0.025 (2)	0.0242 (18)	−0.0035 (15)	0.0143 (16)	−0.0052 (14)
O1	0.0245 (7)	0.0362 (9)	0.0220 (7)	−0.0004 (6)	0.0166 (7)	−0.0002 (6)
O2	0.0234 (7)	0.0315 (9)	0.0153 (7)	−0.0010 (6)	0.0094 (6)	−0.0052 (6)
O3	0.0247 (8)	0.0209 (8)	0.0265 (8)	0.0012 (6)	−0.0012 (7)	0.0038 (6)
O4	0.0286 (7)	0.0152 (7)	0.0164 (7)	−0.0035 (6)	0.0093 (6)	0.0027 (5)
O5	0.0249 (7)	0.0157 (8)	0.0269 (8)	−0.0026 (6)	0.0133 (7)	−0.0063 (6)
O6	0.0240 (7)	0.0214 (8)	0.0427 (9)	−0.0018 (6)	0.0217 (7)	−0.0083 (7)
O7	0.0203 (7)	0.0529 (11)	0.0271 (8)	0.0073 (7)	0.0090 (7)	−0.0051 (7)
O8	0.0375 (9)	0.0247 (9)	0.0297 (8)	−0.0023 (7)	0.0182 (8)	−0.0007 (6)
O9	0.0380 (9)	0.0388 (10)	0.0264 (8)	0.0154 (8)	0.0109 (8)	−0.0026 (7)
O10	0.0254 (7)	0.0183 (7)	0.0179 (7)	−0.0010 (6)	0.0113 (6)	−0.0015 (5)
O11	0.0231 (7)	0.0243 (8)	0.0357 (9)	−0.0002 (6)	0.0117 (7)	−0.0024 (7)
O12	0.0307 (9)	0.0873 (15)	0.0284 (9)	−0.0030 (10)	0.0096 (8)	0.0016 (9)
O13	0.0319 (8)	0.0386 (10)	0.0213 (8)	−0.0010 (7)	0.0034 (7)	−0.0017 (7)
O14	0.0262 (8)	0.0350 (9)	0.0318 (8)	0.0028 (7)	0.0153 (7)	0.0035 (7)
N1	0.0129 (7)	0.0198 (9)	0.0160 (8)	−0.0002 (6)	0.0073 (7)	−0.0001 (6)
N2	0.0128 (7)	0.0172 (9)	0.0141 (8)	0.0001 (6)	0.0062 (7)	0.0005 (6)
N3	0.0153 (7)	0.0164 (9)	0.0132 (8)	−0.0040 (6)	0.0043 (7)	−0.0006 (6)
N4	0.0153 (7)	0.0130 (8)	0.0140 (8)	−0.0013 (6)	0.0058 (7)	−0.0003 (6)
N5	0.0180 (8)	0.0138 (9)	0.0211 (8)	−0.0028 (6)	0.0113 (7)	−0.0015 (6)
N6	0.0163 (7)	0.0144 (8)	0.0182 (8)	−0.0024 (6)	0.0107 (7)	−0.0024 (6)
N7	0.0146 (7)	0.0214 (9)	0.0222 (8)	−0.0026 (6)	0.0101 (7)	−0.0001 (7)
N8	0.0140 (7)	0.0182 (9)	0.0150 (8)	−0.0008 (6)	0.0057 (7)	−0.0008 (6)
N9	0.0152 (8)	0.0214 (9)	0.0161 (8)	−0.0038 (6)	0.0055 (7)	−0.0012 (6)
N10	0.0156 (7)	0.0152 (9)	0.0173 (8)	−0.0027 (6)	0.0078 (7)	−0.0017 (6)
N11	0.0179 (8)	0.0203 (9)	0.0219 (8)	−0.0037 (7)	0.0109 (7)	−0.0025 (7)
N12	0.0166 (8)	0.0172 (9)	0.0197 (8)	−0.0035 (6)	0.0107 (7)	−0.0022 (6)
C1	0.0193 (9)	0.0138 (10)	0.0174 (9)	0.0029 (7)	0.0108 (8)	0.0000 (7)
C2	0.0193 (9)	0.0151 (10)	0.0135 (9)	0.0045 (7)	0.0083 (8)	0.0029 (7)
C3	0.0214 (9)	0.0181 (11)	0.0129 (9)	0.0005 (8)	0.0067 (8)	−0.0010 (7)
C4	0.0219 (9)	0.0140 (10)	0.0146 (9)	0.0017 (7)	0.0102 (8)	−0.0016 (7)
C5	0.0198 (9)	0.0152 (10)	0.0144 (9)	0.0006 (7)	0.0085 (8)	0.0011 (7)
C6	0.0191 (9)	0.0167 (10)	0.0148 (9)	0.0001 (7)	0.0094 (8)	−0.0006 (7)
C7	0.0144 (9)	0.0254 (11)	0.0220 (10)	0.0000 (8)	0.0080 (8)	0.0003 (8)
C8	0.0177 (9)	0.0158 (10)	0.0151 (9)	−0.0025 (7)	0.0051 (8)	−0.0033 (7)
C9	0.0213 (10)	0.0228 (11)	0.0198 (10)	−0.0068 (8)	0.0085 (9)	−0.0051 (8)
C10	0.0218 (9)	0.0175 (11)	0.0208 (10)	−0.0036 (8)	0.0132 (9)	−0.0002 (8)
C11	0.0213 (10)	0.0207 (11)	0.0251 (10)	−0.0047 (8)	0.0143 (9)	0.0005 (8)
C12	0.0148 (9)	0.0197 (11)	0.0207 (10)	0.0024 (7)	0.0084 (8)	0.0013 (8)

### Geometric parameters (Å, º)

**Table:**

Fe1—N5	1.9340 (16)	N1—C1	1.334 (2)
Fe1—N6	1.9398 (15)	N1—N7	1.432 (2)
Fe1—N1	1.9405 (15)	N2—C2	1.346 (2)
Fe1—N4	1.9504 (15)	N2—N8	1.427 (2)
Fe1—N3	1.9516 (16)	N3—C3	1.328 (3)
Fe1—N2	1.9572 (15)	N3—N9	1.424 (2)
Li1—O7	1.957 (4)	N4—C4	1.336 (2)
Li1—O8	1.979 (4)	N4—N10	1.426 (2)
Li1—O9	2.011 (4)	N5—C5	1.343 (2)
Li1—O1	2.178 (4)	N5—N11	1.420 (2)
Li1—N7	2.419 (4)	N6—C6	1.339 (2)
Li2—O10	2.003 (4)	N6—N12	1.424 (2)
Li2—O4	2.020 (4)	N7—C7	1.476 (3)
Li2—O5^i^	2.125 (4)	N7—C11	1.486 (3)
Li2—O2^ii^	2.148 (4)	N8—C12	1.468 (2)
Li2—O1^ii^	2.308 (4)	N8—C8	1.476 (2)
Li2—N10	2.456 (4)	N9—C9	1.458 (3)
Li2—C4	2.732 (4)	N9—C7	1.469 (2)
O1—C1	1.239 (2)	N10—C8	1.469 (2)
O2—C2	1.236 (2)	N10—C10	1.489 (2)
O3—C3	1.244 (2)	N11—C11	1.462 (2)
O4—C4	1.242 (2)	N11—C9	1.480 (3)
O5—C5	1.232 (2)	N12—C10	1.462 (2)
O6—C6	1.232 (2)	N12—C12	1.480 (2)
O7—H00E	0.8601	C1—C2	1.520 (3)
O7—H00F	0.8598	C3—C4	1.512 (3)
O8—H00C	0.8596	C5—C6	1.528 (3)
O8—H00D	0.8599	C7—H01A	0.9800
O9—H00G	0.8602	C7—H01B	0.9800
O9—H00H	0.8595	C8—H00I	0.9800
O10—H00A	0.8651	C8—H00J	0.9800
O10—H00B	0.8656	C9—H01C	0.9800
O11—H00O	0.8595	C9—H01D	0.9800
O11—H00P	0.8598	C10—H00K	0.9800
O12—H00S	0.8604	C10—H00L	0.9800
O12—H00T	0.8600	C11—H01G	0.9800
O13—H00Q	0.8595	C11—H01H	0.9800
O13—H00R	0.8601	C12—H01E	0.9800
O14—H00M	0.8600	C12—H01F	0.9800
O14—H00N	0.8599		
			
N5—Fe1—N6	80.87 (6)	C6—N6—Fe1	117.50 (12)
N5—Fe1—N1	87.19 (7)	N12—N6—Fe1	121.81 (11)
N6—Fe1—N1	159.22 (7)	N1—N7—C7	110.58 (15)
N5—Fe1—N4	158.28 (7)	N1—N7—C11	106.97 (14)
N6—Fe1—N4	86.67 (7)	C7—N7—C11	109.38 (15)
N1—Fe1—N4	109.51 (7)	N1—N7—Li1	101.98 (13)
N5—Fe1—N3	86.93 (7)	C7—N7—Li1	110.93 (14)
N6—Fe1—N3	108.74 (7)	C11—N7—Li1	116.64 (14)
N1—Fe1—N3	87.37 (7)	N2—N8—C12	110.73 (14)
N4—Fe1—N3	80.29 (6)	N2—N8—C8	109.18 (14)
N5—Fe1—N2	110.08 (7)	C12—N8—C8	109.81 (15)
N6—Fe1—N2	87.75 (6)	N3—N9—C9	111.01 (15)
N1—Fe1—N2	80.43 (6)	N3—N9—C7	108.86 (14)
N4—Fe1—N2	86.97 (6)	C9—N9—C7	110.30 (16)
N3—Fe1—N2	158.35 (7)	N4—N10—C8	110.79 (14)
O7—Li1—O8	110.66 (19)	N4—N10—C10	107.59 (14)
O7—Li1—O9	91.73 (17)	C8—N10—C10	109.30 (14)
O8—Li1—O9	105.70 (18)	N4—N10—Li2	96.45 (13)
O7—Li1—O1	86.84 (15)	C8—N10—Li2	115.13 (14)
O8—Li1—O1	111.20 (18)	C10—N10—Li2	116.61 (14)
O9—Li1—O1	141.0 (2)	N5—N11—C11	111.48 (15)
O7—Li1—N7	149.1 (2)	N5—N11—C9	108.65 (15)
O8—Li1—N7	98.46 (16)	C11—N11—C9	110.05 (15)
O9—Li1—N7	90.03 (15)	N6—N12—C10	111.91 (15)
O1—Li1—N7	72.83 (12)	N6—N12—C12	108.65 (14)
O10—Li2—O4	98.61 (16)	C10—N12—C12	109.73 (15)
O10—Li2—O5^i^	91.62 (15)	O1—C1—N1	128.60 (18)
O4—Li2—O5^i^	87.79 (15)	O1—C1—C2	120.30 (17)
O10—Li2—O2^ii^	168.6 (2)	N1—C1—C2	111.04 (16)
O4—Li2—O2^ii^	92.56 (15)	O2—C2—N2	128.98 (18)
O5^i^—Li2—O2^ii^	91.13 (14)	O2—C2—C1	119.85 (16)
O10—Li2—O1^ii^	91.53 (14)	N2—C2—C1	111.16 (16)
O4—Li2—O1^ii^	163.8 (2)	O3—C3—N3	128.68 (18)
O5^i^—Li2—O1^ii^	104.64 (15)	O3—C3—C4	120.46 (17)
O2^ii^—Li2—O1^ii^	77.06 (12)	N3—C3—C4	110.85 (16)
O10—Li2—N10	93.96 (15)	O4—C4—N4	126.82 (18)
O4—Li2—N10	72.34 (12)	O4—C4—C3	121.36 (17)
O5^i^—Li2—N10	159.95 (18)	N4—C4—C3	111.81 (16)
O2^ii^—Li2—N10	87.18 (13)	O4—C4—Li2	43.45 (12)
O1^ii^—Li2—N10	94.45 (14)	N4—C4—Li2	86.89 (13)
O10—Li2—C4	112.06 (15)	C3—C4—Li2	154.14 (15)
O4—Li2—C4	25.02 (7)	O5—C5—N5	127.19 (18)
O5^i^—Li2—C4	107.80 (15)	O5—C5—C6	121.85 (17)
O2^ii^—Li2—C4	77.53 (12)	N5—C5—C6	110.96 (16)
O1^ii^—Li2—C4	138.76 (16)	O6—C6—N6	127.65 (18)
N10—Li2—C4	52.36 (9)	O6—C6—C5	121.43 (17)
C1—O1—Li1	111.59 (16)	N6—C6—C5	110.92 (15)
C1—O1—Li2^ii^	107.21 (15)	N9—C7—N7	113.96 (15)
Li1—O1—Li2^ii^	129.77 (15)	N9—C7—H01A	108.8
C2—O2—Li2^ii^	113.40 (15)	N7—C7—H01A	108.8
C4—O4—Li2	111.52 (16)	N9—C7—H01B	108.8
C5—O5—Li2^iii^	116.16 (15)	N7—C7—H01B	108.8
Li1—O7—H00E	119.2	H01A—C7—H01B	107.7
Li1—O7—H00F	123.7	N10—C8—N8	113.91 (15)
H00E—O7—H00F	104.5	N10—C8—H00I	108.8
Li1—O8—H00C	123.3	N8—C8—H00I	108.8
Li1—O8—H00D	128.7	N10—C8—H00J	108.8
H00C—O8—H00D	104.5	N8—C8—H00J	108.8
Li1—O9—H00G	127.6	H00I—C8—H00J	107.7
Li1—O9—H00H	124.6	N9—C9—N11	113.01 (16)
H00G—O9—H00H	104.5	N9—C9—H01C	109.0
Li2—O10—H00A	109.3	N11—C9—H01C	109.0
Li2—O10—H00B	109.8	N9—C9—H01D	109.0
H00A—O10—H00B	104.2	N11—C9—H01D	109.0
H00O—O11—H00P	104.5	H01C—C9—H01D	107.8
H00S—O12—H00T	104.5	N12—C10—N10	113.43 (15)
H00Q—O13—H00R	104.5	N12—C10—H00K	108.9
H00M—O14—H00N	104.5	N10—C10—H00K	108.9
C1—N1—N7	114.41 (15)	N12—C10—H00L	108.9
C1—N1—Fe1	118.07 (12)	N10—C10—H00L	108.9
N7—N1—Fe1	122.96 (11)	H00K—C10—H00L	107.7
C2—N2—N8	113.54 (15)	N11—C11—N7	113.76 (15)
C2—N2—Fe1	116.37 (12)	N11—C11—H01G	108.8
N8—N2—Fe1	121.41 (11)	N7—C11—H01G	108.8
C3—N3—N9	114.73 (15)	N11—C11—H01H	108.8
C3—N3—Fe1	117.61 (12)	N7—C11—H01H	108.8
N9—N3—Fe1	121.70 (12)	H01G—C11—H01H	107.7
C4—N4—N10	112.84 (15)	N8—C12—N12	113.46 (15)
C4—N4—Fe1	116.34 (12)	N8—C12—H01E	108.9
N10—N4—Fe1	123.19 (11)	N12—C12—H01E	108.9
C5—N5—N11	113.94 (15)	N8—C12—H01F	108.9
C5—N5—Fe1	117.46 (13)	N12—C12—H01F	108.9
N11—N5—Fe1	122.00 (12)	H01E—C12—H01F	107.7
C6—N6—N12	114.37 (15)		
			
C1—N1—N7—C7	147.87 (16)	Fe1—N4—C4—O4	−162.81 (15)
Fe1—N1—N7—C7	−56.60 (19)	N10—N4—C4—C3	167.76 (14)
C1—N1—N7—C11	−93.11 (18)	Fe1—N4—C4—C3	17.16 (19)
Fe1—N1—N7—C11	62.43 (18)	N10—N4—C4—Li2	−30.62 (15)
C1—N1—N7—Li1	29.85 (19)	Fe1—N4—C4—Li2	178.77 (11)
Fe1—N1—N7—Li1	−174.61 (12)	O3—C3—C4—O4	−18.6 (3)
C2—N2—N8—C12	155.31 (15)	N3—C3—C4—O4	160.73 (17)
Fe1—N2—N8—C12	−58.22 (18)	O3—C3—C4—N4	161.42 (18)
C2—N2—N8—C8	−83.67 (18)	N3—C3—C4—N4	−19.2 (2)
Fe1—N2—N8—C8	62.80 (17)	O3—C3—C4—Li2	27.6 (4)
C3—N3—N9—C9	149.01 (16)	N3—C3—C4—Li2	−153.0 (3)
Fe1—N3—N9—C9	−58.87 (18)	Li2^iii^—O5—C5—N5	−104.0 (2)
C3—N3—N9—C7	−89.40 (19)	Li2^iii^—O5—C5—C6	75.8 (2)
Fe1—N3—N9—C7	62.73 (18)	N11—N5—C5—O5	−14.6 (3)
C4—N4—N10—C8	154.71 (15)	Fe1—N5—C5—O5	−166.75 (16)
Fe1—N4—N10—C8	−57.00 (18)	N11—N5—C5—C6	165.57 (15)
C4—N4—N10—C10	−85.87 (17)	Fe1—N5—C5—C6	13.4 (2)
Fe1—N4—N10—C10	62.42 (17)	N12—N6—C6—O6	−14.4 (3)
C4—N4—N10—Li2	34.70 (17)	Fe1—N6—C6—O6	−167.09 (16)
Fe1—N4—N10—Li2	−177.01 (12)	N12—N6—C6—C5	165.63 (14)
C5—N5—N11—C11	151.14 (16)	Fe1—N6—C6—C5	13.0 (2)
Fe1—N5—N11—C11	−58.11 (19)	O5—C5—C6—O6	−16.4 (3)
C5—N5—N11—C9	−87.42 (19)	N5—C5—C6—O6	163.45 (18)
Fe1—N5—N11—C9	63.32 (18)	O5—C5—C6—N6	163.53 (17)
C6—N6—N12—C10	149.74 (16)	N5—C5—C6—N6	−16.6 (2)
Fe1—N6—N12—C10	−58.92 (18)	N3—N9—C7—N7	−67.7 (2)
C6—N6—N12—C12	−88.94 (18)	C9—N9—C7—N7	54.3 (2)
Fe1—N6—N12—C12	62.40 (17)	N1—N7—C7—N9	64.7 (2)
Li1—O1—C1—N1	−13.6 (3)	C11—N7—C7—N9	−52.8 (2)
Li2^ii^—O1—C1—N1	−161.0 (2)	Li1—N7—C7—N9	177.14 (15)
Li1—O1—C1—C2	163.46 (17)	N4—N10—C8—N8	64.37 (19)
Li2^ii^—O1—C1—C2	16.0 (2)	C10—N10—C8—N8	−54.02 (19)
N7—N1—C1—O1	−14.7 (3)	Li2—N10—C8—N8	172.47 (14)
Fe1—N1—C1—O1	−171.54 (16)	N2—N8—C8—N10	−67.39 (19)
N7—N1—C1—C2	168.01 (15)	C12—N8—C8—N10	54.19 (19)
Fe1—N1—C1—C2	11.2 (2)	N3—N9—C9—N11	66.1 (2)
Li2^ii^—O2—C2—N2	−175.26 (19)	C7—N9—C9—N11	−54.7 (2)
Li2^ii^—O2—C2—C1	6.0 (2)	N5—N11—C9—N9	−67.47 (19)
N8—N2—C2—O2	−13.0 (3)	C11—N11—C9—N9	54.8 (2)
Fe1—N2—C2—O2	−161.26 (17)	N6—N12—C10—N10	65.88 (19)
N8—N2—C2—C1	165.79 (15)	C12—N12—C10—N10	−54.8 (2)
Fe1—N2—C2—C1	17.5 (2)	N4—N10—C10—N12	−65.81 (19)
O1—C1—C2—O2	−16.8 (3)	C8—N10—C10—N12	54.6 (2)
N1—C1—C2—O2	160.70 (17)	Li2—N10—C10—N12	−172.70 (14)
O1—C1—C2—N2	164.26 (17)	N5—N11—C11—N7	66.5 (2)
N1—C1—C2—N2	−18.2 (2)	C9—N11—C11—N7	−54.2 (2)
N9—N3—C3—O3	−14.3 (3)	N1—N7—C11—N11	−66.78 (19)
Fe1—N3—C3—O3	−167.66 (17)	C7—N7—C11—N11	53.0 (2)
N9—N3—C3—C4	166.41 (15)	Li1—N7—C11—N11	179.90 (15)
Fe1—N3—C3—C4	13.1 (2)	N2—N8—C12—N12	66.67 (19)
Li2—O4—C4—N4	−27.3 (3)	C8—N8—C12—N12	−54.0 (2)
Li2—O4—C4—C3	152.74 (17)	N6—N12—C12—N8	−67.94 (19)
N10—N4—C4—O4	−12.2 (3)	C10—N12—C12—N8	54.7 (2)

Symmetry codes: (i) *x*, *y*+1, *z*; (ii) −*x*+1, −*y*+1, −*z*+1; (iii) *x*, *y*−1, *z*.

### Hydrogen-bond geometry (Å, º)

**Table:**

*D*—H···*A*	*D*—H	H···*A*	*D*···*A*	*D*—H···*A*
O7—H00*E*···O10^ii^	0.86	1.87	2.715 (2)	167
O7—H00*F*···O8^iv^	0.86	2.08	2.921 (2)	164
O8—H00*C*···O14^v^	0.86	1.90	2.759 (2)	175
O8—H00*D*···O13^vi^	0.86	1.97	2.812 (2)	167
O9—H00*G*···O11^vi^	0.86	1.97	2.827 (2)	176
O9—H00*H*···O14^vii^	0.86	2.02	2.881 (2)	177
O10—H00*A*···O6^i^	0.87	2.00	2.768 (2)	147
O10—H00*B*···O4^viii^	0.87	1.97	2.8301 (18)	178
O11—H00*O*···O3	0.86	2.05	2.844 (2)	154
O12—H00*S*···O11	0.86	1.97	2.828 (2)	177
O13—H00*Q*···O12^vi^	0.86	1.88	2.736 (3)	177
O13—H00*R*···O3	0.86	1.93	2.767 (2)	165
O14—H00*M*···O6	0.86	1.98	2.784 (2)	155
O14—H00*N*···O13^viii^	0.86	2.01	2.867 (2)	176

Symmetry codes: (i) *x*, *y*+1, *z*; (ii) −*x*+1, −*y*+1, −*z*+1; (iv) −*x*+1/2, −*y*+1/2, −*z*+1; (v) −*x*+1, −*y*, −*z*+1; (vi) −*x*+1/2, *y*−1/2, −*z*+1/2; (vii) *x*−1/2, *y*+1/2, *z*; (viii) −*x*+1, *y*, −*z*+1/2.
